# Supplementary material for: EnzML: multi-label prediction of enzyme classes using InterPro signatures
Source: BMC Bioinformatics. 2012 Apr 25;13:61. doi: 10.1186/1471-2105-13-61 (PMC3483700; doi:10.1186/1471-2105-13-61)
Supplement: Addtional file 5 — The Java code to format the data files, evaluate and predict. The file enzml_java_code.tar.gz contains the Java code used to format database data to ARFF and XML formats, to execute cross and train-test (jackknife) evaluations and to record evaluation results to database. More information is included in the readme.txt file and the Javadoc files. The code can be used with a MySQL database. To use a different database software, other JDBC drivers might be required. [file 1471-2105-13-61-S5.gz › java_code/enzml2011/doc/uk/ac/ed/inf/taweka/mulan/learn/MulanArffRecord.html]

MulanArffRecord


---


|  |  |  |
| --- | --- | --- |
| **Overview** \| **Package** \| **Class** \| **Use** \| **Tree** \| **Deprecated** \| **Index** \| **Help** | |  |
| **PREV CLASS**   **NEXT CLASS** | **FRAMES**    **NO FRAMES**     **All Classes** |  |
| SUMMARY: NESTED \| FIELD \| CONSTR \| METHOD | DETAIL: FIELD \| CONSTR \| METHOD |  |


---


## uk.ac.ed.inf.taweka.mulan.learn Class MulanArffRecord

```
java.lang.Object
  uk.ac.ed.inf.taweka.Initialised
      uk.ac.ed.inf.taweka.mulan.learn.MulanArffRecord
```

**All Implemented Interfaces:**: Initialisable

---

``` public class MulanArffRecord extends Initialised ```

Checks that an ARFF record read from the database contains valid parameters
to read an ARFF file.

**Author:**
:   luna.deferrari[at]ed.ac.uk

---

**Field Summary**

**Fields inherited from class uk.ac.ed.inf.taweka.Initialised**

`m_initialisationIsCorrect`


**Constructor Summary**

`MulanArffRecord(int arffRecordId,
java.lang.String arffRecordDbPropsPath)`


| **Method Summary** | |
| --- | --- |
| `java.lang.String` | `deriveXmlPathFromArff(java.lang.String arffProps)` |
| `java.lang.String` | `getArffDbConnPath()` |
| `java.lang.String` | `getArffFileName()` |
| `int` | `getArffId()` |
| `java.lang.String` | `getXmlFileName()` |

**Methods inherited from class uk.ac.ed.inf.taweka.Initialised**

`initialisationIsCorrect, setInitialisationIsCorrect`

**Methods inherited from class java.lang.Object**

`equals, getClass, hashCode, notify, notifyAll, toString, wait, wait, wait`

**Constructor Detail**

### MulanArffRecord

```
public MulanArffRecord(int arffRecordId,
                       java.lang.String arffRecordDbPropsPath)
```


**Method Detail**

### deriveXmlPathFromArff

```
public java.lang.String deriveXmlPathFromArff(java.lang.String arffProps)
```

---


### getArffDbConnPath

```
public java.lang.String getArffDbConnPath()
```

---


### getArffFileName

```
public java.lang.String getArffFileName()
```

---


### getArffId

```
public int getArffId()
```

---


### getXmlFileName

```
public java.lang.String getXmlFileName()
```


---


|  |  |  |
| --- | --- | --- |
| **Overview** \| **Package** \| **Class** \| **Use** \| **Tree** \| **Deprecated** \| **Index** \| **Help** | |  |
| **PREV CLASS**   **NEXT CLASS** | **FRAMES**    **NO FRAMES**     **All Classes** |  |
| SUMMARY: NESTED \| FIELD \| CONSTR \| METHOD | DETAIL: FIELD \| CONSTR \| METHOD |  |


---
